# Chemical Characterization, Antioxidant and Antimicrobial Properties of Goji Berries Cultivated in Serbia

**DOI:** 10.3390/foods9111614

**Published:** 2020-11-06

**Authors:** Tijana Ilić, Margarita Dodevska, Mirjana Marčetić, Dragana Božić, Igor Kodranov, Bojana Vidović

**Affiliations:** 1 Department of Bromatology, Faculty of Pharmacy, University of Belgrade, 11221 Belgrade, Serbia; tijana.ilic@pharmacy.bg.ac.rs; 2 Institute of Public Health of Serbia “Dr Milan Jovanović Batut”, Center for Hygiene and Human Ecology, 11000 Belgrade, Serbia; margarita_dodevska@batut.org.rs; 3 Department of Pharmacognosy, Faculty of Pharmacy, University of Belgrade, 11221 Belgrade, Serbia; mirjana.marcetic@pharmacy.bg.ac.rs; 4 Department of Microbiology and Immunology, Faculty of Pharmacy, University of Belgrade, 11221 Belgrade, Serbia; dragana.bozic@pharmacy.bg.ac.rs; 5 Department of Analytical Chemistry, Faculty of Chemistry, University of Belgrade, 11000 Belgrade, Serbia; ikodranov@chem.bg.ac.rs

**Keywords:** goji, nutritional composition, dietary fiber, microelements, antioxidant activities, antimicrobial properties

## Abstract

Since the fruits of *Lycium* L. species (*Fructus lycii*, goji berries) are promoted as a “superfood” with plenty of health benefits, there is extensive research interest in their nutritional and phytochemical composition. In the present study, the nutritional value, minerals, fatty acid composition, and bioactive compounds of *L. barbarum* L., red, yellow, and black goji berry (*L. ruthenicum* Murray.) cultivated in Serbia were investigated. Antioxidant and antimicrobial properties of their methanol extracts were assessed. Red goji berry had the highest content of fats, dietary fiber, iron, total carotenoids, and 2-*O*-β-d-glucopyranosyl-l-ascorbic acid (AA-2βG). The yellow goji berry extract showed the highest level of flavonoids and the most prominent antimicrobial (especially against Gram-negative bacteria) properties. The highest total phenolic content and the most potent antioxidant activity were observed for the extract of black goji berry. Therefore, all goji berries could be a valuable source of bioactive compounds in the food and pharmaceutical industry.

## 1. Introduction

The genus *Lycium* (Solanaceae) comprises around 100 species, widely distributed in arid to semi-arid regions of North and South America, Africa, and Eurasia. The different plant parts of many species of this genus, including fruits, leaves, young shoots, and root bark, have been consumed as part of a traditional diet and used for medicinal purposes [1]. For more than 2000 years, *Lycium* species have been used in traditional Chinese medicine to treat various diseases, including blurry vision, cough, asthma, diabetes, kidney failure, infertility, and nervous fatigue [1,2]. So far, most of the literature data on phytochemical composition, effectiveness, and safety refer to the *L. barbarum* L. and *L. chinense* Mill. [3]. Hence, recent studies have been focused on other *Lycium* species, including *L. ruthenicum* Murray [4,5,6,7].

Apart from traditional use in China and other Asian countries, fruits of *Lycium* species, *Fructus lycii*, also named as wolfberry or goji berries, have become popular in the global market as a “superfood” [8]. Namely, goji berries, which are characterized by attractive colors (yellow, bright orange to red (*L. barbarum*) or black (*L. ruthenicum*)), sweet and tangy flavors, high nutritive properties and high content of bioactive compounds with a positive impact on human health, have attracted much attention as a potential functional food [9].

Based on the results of many studies, goji berries have been shown to possess antioxidant, immunomodulatory, anticancer, lipid-lowering, and hypoglycemic effects; thus, the incorporation of goji berries into a regular diet may help to prevent many aging-related diseases [2,10]. These health-promoting properties of goji berries are attributed to polysaccharides, carotenoids, polyphenols, and other bioactive compounds [11]. In addition to nutritional and health properties, the presence of these functional compounds make goji berries and derived products suitable for alternative applications, such as improving the stability or sensory quality of food and cosmetic products [12,13,14,15].

Recently, due to growing demand, the cultivation of *Lycium* species has become widespread among European countries, including Serbia. However, the literature data on the chemical composition and biological activities of goji berries cultivated in Serbia are scarce [16]. Therefore, the current study’s aims were to analyze and compare the nutritional value, bioactive compounds, antioxidant and antimicrobial potentials of two varieties of *L. barbarum* L., red (“No. 1”) and yellow (“Amber Sweet Goji”) goji berry, and black goji berry (*L. ruthenicum* Murr.) cultivated in Serbia. According to our knowledge, no study has been carried out to assess and compare the different varieties of *L. barbarum* in Serbia. Additionally, this study reports the chemical composition and biological activities of black goji berry originated from Serbia for the first time.

## 2. Materials and Methods

### 2.1. Samples

Three different samples (red, yellow, and black) of *Lycium* species fruits, commonly known as goji, were collected from a private plantation “Ljuba i sinovi” in Niš, southern Serbia (43°18′58.5″ N 21°49′23.5″ E). Red and yellow goji fruits samples corresponded to *Lycium barbarum* L. varieties, “No.1” and “Amber Sweet Goji”, respectively, and black goji fruits to *Lycium ruthenicum* Murray. The fresh mature goji berries were manually harvested in August 2019 from the three-year-old goji plants; from three plants for each replication. The nutritional and physicochemical analysis was done using fresh samples or stored at 4 °C for a few days. Parts of the samples were dried at 60 °C for 24 h, ground in a laboratory mill and stored at 4 °C for further use. For the determination of phenolic compounds, antioxidant assays, and antimicrobial analysis, goji berry fruit samples were homogenized and methanol extracts were prepared. Briefly, after addition methanol/water (80/20, *v*/*v*), the mixture was sonicated for 15 min twice and left at room temperature in darkness for 24 h. After that, the extracts were filtered through Whatman No.4 paper, evaporated under vacuum using a rotary evaporator (BUCHI rotavapor R-100, type B100-HB, Flawil, Switzerland), and stored at 4 °C until analyses. The extracts were re-dissolved in an appropriate solvent to a 10 mg/mL concentration before using the quantitative analysis of total phenolic compounds and in vitro bioactivity evaluation.

### 2.2. Nutritional Composition

#### 2.2.1. Nutritional Value

The goji berry samples were analyzed for the moisture, proteins, fat, and ash content using the procedures described by the Association of Official Analytical Chemists (AOAC) [17]. Soluble (SDF) and insoluble dietary fiber (IDF) contents were determined by the enzymatic-gravimetric procedure according to the AOAC method 991.43 [18], following the procedure described by Le et al. [19], using the enzymatic Total Dietary Fiber Assay Kit (K-TDFR; Megazyme International, Wicklow, Ireland). The total fibers (TDF) were the sum of soluble and insoluble dietary fiber contents, and carbohydrate contents were calculated as the residual differences after subtracting other compounds from 100. The results were expressed as g/100 g of fresh weight (FW). The energy values were calculated according to the following equation [20]:

Energy, kJ = [37 × (g fat) +17 (g protein + g carbohydrates) + 8 × (g total fiber)](1)

#### 2.2.2. Fatty Acids

The fatty acid composition was determined by gas chromatography after the conversion of fatty acid glycerides into the corresponding methyl esters, according to the ISO 12966-2 procedure [21]. The analyses were performed using Agilent 7890a GC (Agilent 7890A, Santa Clara, CA, USA) with a flame ionization detector. Separations were made on a DB-5MS fused silica capillary column (60 m × 0.320 mm i.d.) with a film thickness of 1 µm. The fatty acid methyl esters (FAMEs) were identified by comparing the chromatogram’s retention times with a reference mixture of FAMEs (Supelco 37 component FAME mix). Individual FAMEs were quantified and the fatty acid profiles were expressed in (% *w*/*w*) of saturated (SFA), monounsaturated (MUFA), polyunsaturated (PUFA) and unsaturated (UFA) fatty acids. The PUFA/SFA ratio, as well as atherogenic index (AI) and oxidisability (Cox) were calculated as described by Ulbricht and Southgate [22] and Fatemi and Hammond [23], respectively:

AI = [(4 × C14:0 + C16:0 + C18:0)**/**(∑ MUFA + ∑ n−6 PUFA + ∑ n−3 PUFA)] (2)

Cox = [(C18:1 + 10.3 × C18:2 + 21.6 × C18:3)/100](3)

#### 2.2.3. Mineral Composition

The analysis was performed by inductively coupled plasma optical emission spectrometry (ICP-OES) using a Thermo Scientific iCAP 6500 Duo (Thermo Fisher Scientific, Cambridge, UK) after microwave digestion described by Yossa Nzeuwa et al. [24]. Mineral contents were quantified against standard solutions of known concentrations, which were analyzed simultaneously, and results were expressed as mg per 100 g of fresh goji berry fruits.

### 2.3. Physicochemical Analysis

The pH values were measured directly in the homogenized samples using a pH meter (Wissenschaftlich-Technische Werkstatten, Weilheim, Germany). Total soluble solids (TSS), expressed as °Brix, were determined using a manual temperature-compensated refractometer (HI 96,811 Digital Brix Refractometer, Hanna Instruments^®^, Woonsocket, RI, USA) according to the standard method [25]. Titratable acidity (TA) determination was performed according to SRPS EN 12147:2005 and expressed as a percentage of citric acid in fresh goji berry [26]. The ratio TSS/TA was calculated.

### 2.4. Determination of Bioactive Compounds

#### 2.4.1. 2-*O*-β-d-glucopyranosyl-l-Ascorbic Acid (AA-2βG)

Determination of AA-2βG, a natural ascorbic acid derivative, from goji berry samples was carried out using Agilent 1200 series high-performance liquid chromatography (HPLC) (Agilent Technologies, Santa Clara, CA, USA) with diode array detection (DAD) according to a previously described procedure by Trang et al. [27]. The method was gradient elution with a flow rate of 1.3 mL/min, and the mobile phase was water with 0.1% trifluoroacetic acid (TFA) and acetonitrile with 0.1% TFA. The separation was performed on a Zorbax Eclipse XDB column (150 mm × 4.6 mm, 5µm particle size). Quantification was done on 245 nm wavelength, and results were expressed as mg AA-2βG/100 g FW.

#### 2.4.2. Total Carotenoid Contents (TCC)

The total carotenoid contents (TCC) were extracted from the goji berry samples and analyzed spectrophotometrically, according to Nagata and Yamashita [28]. Briefly, after repeating the extraction with a solution of acetone: hexane (4:6 *v*/*v*) several times, the absorbances of the combined supernatants were measured at 453, 505, 645, and 663 nm by a UV-Visible spectrophotometer (Thermo Scientific Evolution 201, Waltham, MA, USA). The results were presented as mg TCC per 100 g of fresh goji berry fruits (mg TCC/100 g).

#### 2.4.3. Phenolic Compounds

##### Total Phenolic Content (TPC)

The total polyphenolic content (TPC) was determined using the Folin-Ciocalteu reagent, according to Singleton and Rossi method [29]. Standard of gallic acid (Acros Organics, Geel, Belgium) 0.1 g/L was used to obtain the standard curve with a 10–80 µg/mL range. The absorbance was read at 765 nm on a spectrophotometer uniSPEC 2 (LLG Labware, Turnov, Czech Republic). The results were expressed as milligrams of gallic acid equivalents (GAE) per 100 g of fresh goji berry fruits (mg GAE/100 g).

##### Total Flavonoid Content (TFC)

The quantitative analysis of total flavonoids was performed by a spectrophotometric method using aluminum chloride, as described in the 7th European Pharmacopoeia [30]. The absorbance was measured at 425 nm (uniSPEC 2, LLG Labware, Turnov, Czech Republic). The percentages of total flavonoids were expressed as mg of hyperoside equivalent (HE) per 100 g of fresh goji berries (mg HE/100 g).

##### Total Anthocyanin Content (TAcy)

The total content of anthocyanins was estimated using the method following the European Pharmacopoeia procedure [30]. Spectrophotometric measurement was performed at 528 nm (uniSPEC 2, LLG Labware, Turnov, Czech Republic), and the content of anthocyanins was expressed as milligram of cyanidin 3-O-glucoside equivalent per 100 g of fresh goji berry fruits (mg C3G/100 g).

##### Total Tannin Content

The determination of tannin content was performed indirectly using the skin powder precipitation method following the procedure described in the 7th European Pharmacopoeia [30]. Tannin content was expressed as mg of pyrogallol equivalents per 100 g of fresh goji berry fruits (mg PYE/100 g).

### 2.5. Antioxidant Activity

Five different in vitro assays: ferric reducing antioxidant power (FRAP), cupric ion reducing antioxidant capacity (CUPRAC), 2,2’-diphenyl-1-picrylhydrazyl radical scavenging activity (DPPH), 2,2’-azino-bis(3-ethylbenzothiazoline)-6-sulphonic acid radical cation scavenging activity (ABTS), and β-carotene bleaching inhibition were used to estimate antioxidant activity.

#### 2.5.1. FRAP Assay

This assay was performed following the method of Benzie and Strain [31]. Trolox (Sigma Aldrich) was used as the standard (100–800 µM) to construct the calibration curve. Absorbance readings were taken at 593 nm (uniSPEC 2, LLG Labware, Turnov, Czech Republic). The results were expressed as µmol of Trolox equivalents (TE) per 100 g of fresh goji berry fruits (µM TE/100 g).

#### 2.5.2. CUPRAC Assay

The cupric ion reducing antioxidant capacity of goji berry extracts was determined according to the method described by Apak et al. [32]. Absorbance was measured at 450 nm (uniSPEC 2, LLG Labware, Turnov, Czech Republic). A calibration curve was built with Trolox as a standard in the range from 200 to 700 µM and the results were expressed as µM of Trolox equivalents (TE)/100 g of fresh goji berry fruits.

#### 2.5.3. DPPH Assay

The radical scavenging activities of goji berry extracts against DPPH radicals were evaluated using a method described by Brand-Williams et al. [33]. The absorbance readings were taken at 517 nm (uniSPEC 2, LLG Labware, Turnov, Czech Republic), and the standard curve was constructed by Trolox (200–700 µM). The results were expressed as µM of Trolox equivalents (TE) per 100 g of fresh goji berry fruits (µM TE/100 g).

#### 2.5.4. ABTS Assay

The radical scavenging activity of goji berry extracts against ABTS radicals was evaluated using a decolorization assay described by Re et al. [34]. The calibration curve was constructed using a range of 0.2–1.5 mM Trolox. Absorbance was measured at 734 nm (uniSPEC 2, LLG Labware, Turnov, Czech Republic). The results were expressed as mM of Trolox equivalents (TE) per 100 g of fresh goji berry fruits (mM TE/100 g).

#### 2.5.5. Beta-Carotene Bleaching Assay

Antioxidant activity of goji berry samples against lipid peroxyl radical was done according to Reis et al. [35]. Absorbance at 470 nm was immediately measured by uniSPEC 2 Spectrophotometer (LLG Labware, Turnov, Czech Republic) after the addition of a sample to β-carotene/linoleic acid emulsion as well as after the incubation period. The results were expressed as a percentage of β-carotene bleaching inhibition.

#### 2.5.6. Antioxidant Composite Index

An overall antioxidant composite index (ACI) of goji berry fruits was determined based on the previously obtained results using FRAP, CUPRAC, DPPH, ABTS, and beta-carotene bleaching assays. An index value of 100 is assigned to the best score for each assay. The mean value of all five tests was taken as the ACI value of each analyzed sample [36].

### 2.6. Antimicrobial Activity

The antimicrobial activity of goji berry extracts was tested against eight laboratory control strains of microorganisms: three Gram-positive bacteria-*Staphylococcus aureus* subsp. *aureus* Rosenbach (American Type Culture Collection (ATCC) 6538), *Staphylococcus epidermidis* (ATCC 12228) and *Enterococcus faecalis* (ATCC 29212); four Gram-negative bacteria-*Escherichia coli* (ATCC 25922), *Klebsiella pneumoniae* subsp. *Pneumoniae* (National Collections of Industrial, Food and Marine Bacteria (NCIMB) 8267), *Salmonella enterica* subsp. *enterica* serovar Abony (National Collection of Type Cultures (NCTC) 6017) and *Pseudomonas aeruginosa* (ATCC 27853), and one yeast *Candida albicans* (ATCC 24433). Minimal inhibitory concentrations of extracts were determined by the broth microdilution test in 96-well microtiter plates, according to the European Committee for Antimicrobial Susceptibility Testing guidelines [37]. The suspension of microorganisms was made in a saline solution to a density of 0.5 per McFarland standard (Bio-Merieux, Marcy l’Etoile, France). Extracts were dissolved in dimethyl sulfoxide DMSO, and further prepared in concentrations ranging from 125–2000 μg/mL in fresh Mueller-Hinton broth (MHB, Lab M Limited, Bury, UK) for bacteria, or Sabouraud-dextrose broth (SDB, Lab M Limited, Bury, UK) for *C. albicans*. Each concentration was set in duplicate and inoculated with 5 × 10^5^ CFU/mL of microorganisms. For the detection of cell growth and metabolism, MHB was supplemented with 0.05% triphenyltetrazolium chloride (TTC, Sigma-Aldrich, St. Louis, MO, USA). TTC is a redox indicator and a colorless dye that becomes a red metabolite of 1.3.5-triphenyformazan due to cellular dehydrogenase activity. Minimal inhibitory concentrations (MIC) were determined after incubation for 20 h at 35 °C in aerobic conditions as the lowest concentration of extract that inhibits bacterial growth (i.e., shows no visible change in medium color). Positive controls (microorganisms in medium) and negative controls (only medium with extracts) were included in the experiments.

### 2.7. Statistical Analysis

All analyses were done in triplicate. The results were expressed as the mean values and the standard deviations (SD). The differences between the goji berries fruit samples were analyzed using a one-way analysis of variance (ANOVA) and the Tukey HSD test. Statistical analyses were processed using the program SPSS (version 20, Chicago, IL, USA) and a *p* < 0.05 was considered statistically significant.

## 3. Results and Discussion

### 3.1. Nutritional Composition

Results regarding the nutrient content and energy value of the analyzed goji berries are presented in Table 1.

All goji berry fruits were characterized by high moisture content. Black goji fruits had significantly higher moisture content than the other samples (*p* < 0.05), consequently resulting in the lowest energy value. The energy value of black goji berries was 38% and 30% lower compared to the red and yellow goji berries, respectively. In addition to the influence on energy density, the high moisture content limits the shelf-life of fresh goji berry fruits. Therefore, the majority (about 90%) of fresh goji berries are processed into dried goji berries, juice, wine, tea, tinctures, powders, and tablets [2,8].

The fat content of the red goji was significantly higher than that of the other varieties (*p* < 0.05). Proteins and ash contents were not significantly different among goji berry varieties (*p* > 0.05), although slightly higher protein contents were observed in the yellow goji berry fruits. In general, the TDF content in red goji and yellow goji was similar and significantly higher than that of the black goji. These results are in line with previous studies pointing out that dietary fibers are the second macronutrient in goji berries [7,38]. As red and yellow goji berries had more than 3 g TDF per 100 g, they meet the requirement for the “source of fiber” claim [39].

The fatty acid compositions of the studied goji berries are presented in Table 2. The most abundant fatty acids were linoleic (C18:2n-6), oleic (C18:1n-9), palmitic (C16:0), and stearic (C18:0) acid, accounting for about 95% of total fatty acids. Similar results were reported for red goji berry cultivated in Italy [40,41], Greece [42], Turkey [43], North Macedonia [44] and for Chinese black goji berries [24,45].

To the best of our knowledge, there are no previous data regarding the fatty acid composition of yellow goji berry. Despite the similar fatty acid profiles, there were significant differences in the relative percentages of individual fatty acids among the studied goji berries, resulting in different SFA, MUFA, and PUFA contents. The black goji berry had the highest SFA content, followed by red and yellow goji berries. The most abundant SFA in all goji berries was palmitic acid (11.8–20.4%), followed by stearic acid (3.0–6.9%), while the presence of heptadecanoic acid was only detected in red and yellow goji berries. Oleic acid content, a predominant MUFA, varied between 17.1% and 23.6% in black and red goji berry, respectively. All studied goji berries contained more than 50% of linoleic acid. The highest amount of linoleic acid in yellow goji berry (59.38%) corresponded with the highest PUFA content of this variety compared to others.

Considering the fatty acids’ potential health effects, the PUFA/SFA should be more than 0.45 [46]. In this study, the PUFA/SFA ratio for red, yellow, and black goji berry was 2.52, 3.59, and 1.86, respectively, indicating that all goji berries’ lipids could be beneficial for human health. In addition to the highest PUFA/SFA ratio, yellow goji berry was characterized by the lowest value of the atherogenic index (AI). In terms of oxidative stability, the lowest Cox value was determined for black, followed by red and yellow goji berry oils.

The content of macro- and microelements for the studied goji berries is shown in Table 3. Mineral concentrations varied significantly across the different goji berry samples. The decreasing order of minerals in both *L. barbarum* varieties was: K > P > Na > S > Mg > Ca > Fe > Zn > Cu > Mn > Cr > B > Se. The red goji berries had about two times more Ca and Fe than yellow goji berries. On the other hand, the higher concentrations of K and Cu and much lower Na were found in yellow goji than red goji berries.

Considering that both goji berry samples were collected from the same plantation and share the same harvesting and post-harvesting handling procedure for mineral analysis, observed variations in mineral content could be mostly attributed to different goji varieties. The decreasing order of minerals in black goji berries showed some differences, in comparison to other goji berry samples: K > P > Na > Ca > Mg > S > Fe > Zn > B > Mn > Cu > Cr > Se. A similar mineral profile of black goji berries was evidenced in a study conducted by Liu et al. [7]. In this study, except for the Na and Ca, all mineral content in black goji berries was lower than in red and yellow goji berries.

Considering the content of minerals expressed as a percentage of the Recommended Daily Allowance (RDA) values (Table 4), there was evidence that all goji berry samples contained K, P, and Cu levels which were more than 15% of the RDA. The red goji berries contained 15.7% of the RDA for iron and could be recommended as a source of this valuable microelement. In this study, the iron content in red goji berry was about 5-fold higher than found in fresh red goji berries of Italian origin [38,41].

### 3.2. Physicochemical and Bioactive Compounds Analysis

The TSS, TA, their ratio (TSS/TA), and pH values, as important indicators of sensory quality of goji berries, are shown in Table 5. The Brix value, which reflects the TSS content in fruits, ranged from 9.43 to 16.73% with significant differences among analyzed goji berry varieties. The red goji had the highest TSS content, followed by the yellow goji, while the black goji had the lowest TSS level. The TA of analyzed goji berries ranged from 0.70 to 0.89% and the average pH values were 4.56–4.71, with the highest acidity observed for the black goji berry. Our results are comparable to those of Zhang at el. [47], who also found the lowest TSS and the highest TA value in *Lycium ruthenicum* compared to the other genotypes. In this study, the TSS/TA ratios were, on average, two times higher in red and yellow goji than black goji, indicating higher sweetness for *L. barbarum* berries.

**Figure 1:**
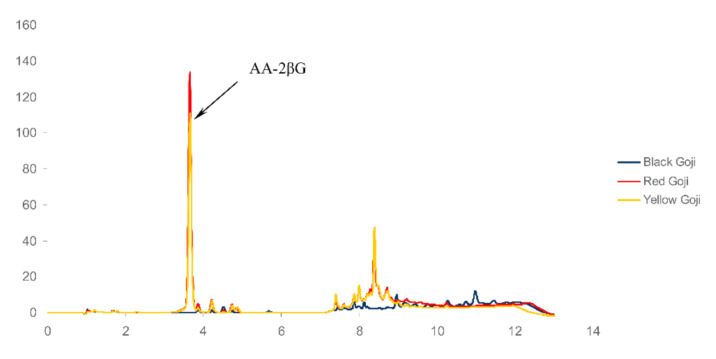
High-performance liquid chromatography (HPLC) chromatogram of goji berry samples at 245 nm. AA-2βG: 2-*O*-β-d-glucopyranosyl-l-ascorbic acid.

The *Lycium* fruits are considered valuable sources of micronutrients, including vitamins, such as thiamin, riboflavin, and vitamin C [9]. Donno et al. [48] showed that average vitamin C content in fresh red goji berries was 48.94 mg/100 g, which makes up approximately 60% of the RDA [20]. Most of the other studies have reported that vitamin C of *L. barbarum* berries ranged from 30 to 60 mg/100 g FW [16,38,41,49,50], depending on the goji berry cultivars and their growing regions. However, some authors found a ten times lower vitamin C content in some goji berry varieties [51,52]. In addition to genotypic differences, pre-harvest climatic conditions and cultural practices, harvesting and post-harvesting handling procedures, including experimental conditions, could also affect the vitamin C content [53]. In addition to L-ascorbic acid, the fruit of *Lycium barbarum* L. contained AA-2βG, documented as pro-vitamin C [54,55] with unique antioxidant activity [56]. AA-2βG is estimated to account for 0.5% of dried goji berry, comparable to the fresh lemons’ ascorbic acid content [54]. Kosińska-Cagnazzo et al. [57] found a range from 0.35 to 2.79 mg/g DW AA-2βG in six goji cultivars from Switzerland, with the highest content of AA-2βG in a cultivar with the highest sugar content. In our study, the AA-2βG content of red goji berry (0.61 mg/g FW; corresponding 2.5 mg/g DW) was higher than in the yellow goji berry (0.48 mg/g FW, corresponding 2.2 mg/g DW), while no AA-2βG was detected in black goji berry. Chromatograms are shown in Figure 1. The obtained results of AA-2βG correspond to the L-ascorbic acid content of 33.4 mg/100 g FW and 24.2 mg/100 g FW for red and yellow goji berry, respectively.

Regarding total carotenoids, red goji berry contained significantly higher amounts compared with yellow goji berry and no TCC content was detected in black goji berry. These results are in agreement with those obtained by Liu et al. [58]. They found that red goji accumulated high carotenoids content (primarily zeaxanthin), while the TCC was undetectable in black goji fruits at the ripe stage. The significant variability in TCC among different *Lycium* species was also previously reported by Zhang et al. [47] and Peng et al. [59].

The black goji berry presented the highest TPC content, which was significantly different from those of the other goji berries. The highest TFC was found in yellow goji, followed by red goji and much lower content in black goji berries.

The tannin content of black goji berry samples was two times higher than that of the red and yellow goji berries. In contrast to black goji berry, anthocyanins were not detected in red and yellow goji berries. Overall, these results indicate that the phenolic content differs significantly among different goji berry varieties, probably reflecting their specific health-promoting effects [60]. In addition, the observed differences in the polyphenol contents compared to the literature data, including previously published data on red goji berry fruit cultivated in north Serbia [16], could be explained by different climate and soil factors that affected the plant development [61,62], as well as extraction procedures that were applied [63].

### 3.3. Antioxidant Activities

The results of the antioxidant activities of methanol goji berry extracts carried out by five different assays are presented in Table 6.

In particular, black goji showed the best results in metal-reducing activity (FRAP, CUPRAC) and radical scavenging activities (DPPH, ABTS). The extracts from red and yellow goji berries had a higher capacity to inhibit lipid peroxidation by β-carotene bleaching assay. Similarly, in the study by Islam et al. [5], black goji berry extracts showed higher antioxidant activity for the FRAP and radical scavenging assays (DPPH, ABTS), compared to the red goji berries. Xin et al. [64] also reported that black goji samples have higher FRAP and DPPH activities than the red goji berries. There is evidence that the main bioactive compounds, phenolics, flavonoids, carotenoids, and polysaccharides, contribute differently to the antioxidant activities of goji berries [47]. Recently, Liu et al. [7] showed that the major contributors for antioxidant activity (DPPH, ABTS, FRAP, and ORAC assays) of black goji berries are phenolic compounds and polysaccharides. Overall, the methanol extract from black goji berry showed the highest ACI values (94.45), indicating that this fruit has nearly 1.7 times higher antioxidant potential than red and yellow goji berries.

### 3.4. Antimicrobial Activities

The investigated goji berries extracts showed mild antimicrobial activity against Gram-positive, Gram-negative bacteria and yeast (Table 7).

The best activity was obtained with yellow goji berry extract, which inhibited the growth of three Gram-negative strains (*K. pneumoniae*, *S. abony* and *P. aeruginosa*) and yeast *C. albicans* at 2 mg/mL. Similarly, previous research showed that hydromethanolic extract of goji berries inhibited the growth of Gram-positive (MIC values 2.5–5 mg/mL) and Gram-negative bacteria (MIC values 2.5–20 mg/mL) [65]. The higher content of bioactive compounds, such as flavonoids, in the yellow goji berry extract, is probably associated with its prominent antibacterial potential compared to other analysed extracts [66]. In addition, Pedro et al. [67] showed that organic goji berry oils and extracts showed higher antimicrobial activity in comparison with samples of conventional fruits, which can be explained by the highest contents of fatty acids and carotenoids. So, the observed antibacterial potential of yellow goji berry could be attributed to the highest UFA and linoleic acid content. The black goji berry extract with high anthocyanin content did not inhibit microorganisms’ growth at the investigated concentrations (0.125–2 mg/mL). Previous research showed that anthocyanins from black goji berries were not digested, but interacted with intestinal microbiota and stimulated fermentation and short-chain fatty acid production [68]. Furthermore, such modulation of the intestinal microbiota, due to a diet rich in anthocyanins, could lead to an anti-inflammatory effect in the gastrointestinal tract [69].

## 4. Conclusions

For the first time, this study offers detailed nutritional value, fatty acids, minerals, bioactive compounds, antioxidant and antimicrobial activity of *Lycium* fructus cultivated in Serbia. Based on the obtained results, it can be concluded that berries of *L. barbarum* L. (“No 1”), red goji, represent sources of dietary fiber, iron, total carotenoids, and provitamin C (AA-2βG). Yellow goji berry, *L. barbarum* (“Amber Sweet Goji”), contained higher PUFAs and linoleic (n-6) content than the other varieties studied. Likewise, yellow goji represented the most abundant flavonoids source and displayed the most prominent antimicrobial (especially against Gram-negative bacteria) properties. The highest total phenolic content, including anthocyanins and the most potent antioxidant activity, was observed for the extract of black goji berry, *L. ruthenicum*. Overall, the favorable results of the nutritional and biological values of goji berries reported in this study may further enhance the cultivation of goji berry in Serbia. A more detailed phytochemical composition analysis of the goji berries would help to further characterize their functional properties.

## Figures and Tables

**Table 1:** Nutritional composition and energy value of the studied goji berry fruits (mean ± SD; n = 3).

	Red Goji	Yellow Goji	Black Goji
Nutritional value (g/100 g FW)			
Moisture	75.32 ± 0.34 ^b^	77.52 ± 0.73 ^b^	84.19 ± 0.36 ^a^
Ash	0.84 ± 0.07 ^a^	0.88 ± 0.09 ^a^	0.63 ± 0.05 ^a^
Fat	1.15 ± 0.08 ^a^	0.83 ± 0.06 ^b^	0.71 ± 0.03 ^b^
Proteins	1.98 ± 0.06 ^a^	2.24 ± 0.30 ^a^	1.68 ± 0.01 ^a^
Total fiber (TDF)	3.63 ± 0.25 ^a^	3.34 ± 0.17 ^a^	2.76 ± 0.21 ^b^
Insoluble fiber (IDF)	2.73 ± 0.16 ^a^	2.68 ± 0.10 ^a^	2.17 ± 0.15 ^b^
Soluble fiber (SDF)	0.90 ± 0.09 ^a^	0.66 ± 0.07 ^b^	0.59 ± 0.08 ^b^
Available carbohydrates	16.93 ± 0.15 ^a^	15.08 ± 0.8 ^b^	9.91 ± 0.29 ^c^
Energy value (kJ/100 g FW)	394 ± 6 ^a^	353 ± 11 ^b^	246 ± 6 ^c^

Different letters in the same row indicate significant differences (*p* < 0.05).

**Table 2:** Fatty acid composition (% of total fatty acids) and lipid indices of the goji berry fruits (mean ± SD; n = 3).

	Red Goji	Yellow Goji	Black Goji
C14:0	0.34 ± 0.02 ^c^	0.49 ± 0.03 ^b^	0.91 ± 0.02 ^a^
C14:1	0.44 ± 0.02 ^b^	0.33 ± 0.01 ^c^	0.71 ± 0.02 ^a^
C16:0	17.64 ± 0.07 ^b^	11.76 ± 0.08 ^c^	20.42 ± 0.04 ^a^
C16:1	0.69 ± 0.04 ^c^	0.70 ± 0.02 ^b^	1.36 ± 0.03 ^a^
C17:0	0.37 ± 0.02 ^a^	0.33 ± 0.03 ^a^	nd
C18:0	3.03 ± 0.06 ^b^	4.25 ± 0.05 ^c^	6.93 ± 0.03 ^a^
C18:1n-9	23.55 ± 0.09 ^a^	21.58 ± 0.08 ^b^	17.13 ± 0.05 ^c^
C18:2n-6	52.08 ± 0.08 ^b^	59.38 ± 0.13 ^a^	49.45 ± 0.03 ^c^
C18:3n-3	1.87 ± 0.11 ^b^	1.19 ± 0.02 ^c^	3.11 ± 0.03 ^a^
∑ SFA	21.38 ± 0.13 ^b^	16.83 ± 0.18 ^c^	28.26 ± 0.08 ^a^
∑ MUFA	24.68 ± 0.03 ^a^	22.61 ± 0.07 ^b^	19.20 ± 0.04 ^c^
∑ PUFA	53.94 ± 0.04 ^b^	60.58 ± 0.11 ^a^	52.55 ± 0.01 ^c^
∑ UFA	78.63 ± 0.01 ^b^	83.18 ± 0.04 ^a^	71.75 ± 0.05 ^c^
PUFA/SFA	2.52 ± 0.01 ^b^	3.59 ± 0.04 ^a^	1.86 ± 0.01 ^c^
AI	0.28 ± 0.00 ^b^	0.22 ± 0.00 ^c^	0.43 ± 0.00 ^a^
Cox	6.00 ± 0.01 ^b^	6.59 ± 0.01 ^a^	5.94 ± 0.00 ^c^

nd-not detected; myristic acid (C14:0); myristoleic acid (C14:1); palmitic acid (C16:0); palmitoleic acid (C16:1); heptadecanoic acid (C17:0); stearic acid (C18:0); oleic acid (C18:1n9); linoleic acid (C18:2n6); linolenic acid (C18:3n3); SFA—saturated fatty acids; MUFA—monounsaturated fatty acids; PUFA—polyunsaturated fatty acids; UFA—unsaturated fatty acids; AI—atherogenic index; Cox—oxidisability value; different letters in the same row indicate significant differences (*p* < 0.05).

**Table 3:** Results of mineral analysis of goji berry fruits, mg/100 g FW (mean ± SD; n = 3).

	Red Goji	Yellow Goji	Black Goji
Na	74.57 ± 1.19 ^b^	51.09 ± 0.30 ^c^	79.55 ± 0.57 ^a^
K	445.12 ± 5.48 ^b^	588.41 ± 0.74 ^a^	336.96 ± 1.55 ^c^
Ca	29.02 ± 0.41 ^b^	15.32 ± 0.12 ^c^	46.33 ± 0.34 ^a^
Mg	29.19 ± 0.43 ^a^	27.15 ± 0.12 ^b^	25.93 ± 0.48 ^c^
P	231.52 ± 3.89 ^a^	213.98 ± 0.96 ^b^	154.76 ± 0.85 ^c^
S	35.86 ± 0.36 ^b^	41.22 ± 0.17 ^a^	22.69 ± 0.14 ^c^
Cu	0.39 ± 0.02 ^b^	0.48 ± 0.01 ^a^	0.16 ± 0.01 ^c^
Zn	0.97 ± 0.02 ^a^	0.91 ± 0.01 ^b^	0.67 ± 0.01 ^c^
Mn	0.25 ± 0.01 ^a^	0.22 ± 0.01 ^b^	0.19 ± 0.01 ^c^
Fe	2.20 ± 0.03 ^a^	1.15 ± 0.01 ^b^	0.70 ± 0.01 ^c^
Cr	0.02 ± 0.01 ^b^	0.03 ± 0.01 ^a^	0.01 ± 0.01 ^c^
Co	nd	0.24 ± 0.01	0.02 ± 0.00
Se	0.006 ± 0.00 ^a^	0.004 ± 0.00 ^a^	0.002 ± 0.00 ^a^
B	0.37 ± 0.12 ^a^	0.37 ± 0.01 ^a^	0.25 ± 0.01 ^b^

nd—not detected; different letters in the same row indicate significant differences (*p* < 0.05).

**Table 4:** Percentage contribution to the recommended daily allowance (RDA) of minerals for 100 g of goji berry fruits related to Regulation (EU) No. 1169/2011.

	RDA (mg/Day)	Red Goji	Yellow Goji	Black Goji
K	2000	22.3	29.4	16.8
Ca	800	3.6	1.9	5.8
Mg	375	7.8	7.2	6.9
P	700	33.1	30.5	22.1
Cu	1	38.9	47.8	15.6
Zn	10	9.7	9.1	6.7
Mn	2	12.7	11.1	9.9
Fe	14	15.7	8.2	5.0
Cr (µg)	40	50.8	75.8	31.7
Se (µg)	55	9.1	6.7	4.2

**Table 5:** Titratable acidity (TA), pH, total soluble solids (TSS), TSS/TA and bioactive compounds in analyzed goji berry fruits.

	Red Goji	Yellow Goji	Black Goji
TA (% citric acid)	0.70 ± 0.07 ^a^	0.70 ± 0.06 ^a^	0.89 ± 0.08 ^b^
pH	4.71 ± 0.04 ^a^	4.71 ± 0.02 ^a^	4.56 ± 0.01 ^b^
TSS (°Brix)	16.73 ± 0.12 ^a^	14.97 ± 0.06 ^b^	9.43 ± 0.06 ^c^
TSS/TA	22.44 ^a^	22.54 ^a^	10.69 ^b^
AA-2βG (mg/100 g)	60.84 ± 3.23 ^a^	48.85 ± 3.43 ^b^	nd
TCC (mg/100 g)	41.71 ± 1.23 ^a^	3.60 ± 0.02 ^b^	nd
TPC (mg GAE/100 g)	162.4 ± 11.5 ^a^	176.3 ± 13.0 ^a^	295.7 ± 18.8 ^b^
TFC (mg HE/100 g)	214.2 ± 28.6 ^b^	335.5 ± 27.1 ^a^	27.40 ± 4.76 ^c^
TAcy (mg C3 G/100 g)	nd	nd	196.0 ± 10.5
Total tannins (mg PYE/100 g)	0.31 ± 0.02 ^b^	0.33 ± 0.02 ^b^	0.63 ± 0.03 ^a^

Nd—not detected; TA—titratable acidity; TSS—total soluble solids; AA-2βG: 2-*O*-β-d-glucopyranosyl-l-ascorbic acid; TCC—total carotenoid content; TPC—total phenolic content; TFC—total flavonoid content; TAcy—total anthocyanin content; different letters in the same row indicate significant differences (*p* < 0.05).

**Table 6:** Antioxidant activity of studied goji berry fruits (mean ± SD; n = 3).

	Red Goji	Yellow Goji	Black Goji
FRAP (µmol TE/100 g)	532.4 ± 21.1 ^b^	578.5 ± 21.8 ^b^	1943.9 ± 18.3 ^a^
CUPRAC (µmol TE/100 g)	616.7 ± 3.3 ^b^	742.7 ± 8.9 ^b^	1057.4 ± 7.1 ^a^
DPPH (µmol TE/100 g)	452.6 ± 3.8 ^b^	443.6 ± 4.1 ^b^	1022.5 ± 3.6 ^a^
ABTS (mmol TE/100 g)	12.9 ± 0.7 ^b^	14.4 ± 0.9 ^b^	28.4 ± 1.5 ^a^
β-carotene bleaching inhibition (%)	22.0 ± 2.6 ^a^	18.6 ± 1.7 ^a, b^	15.9 ± 2.5 ^b^
ACI (%)	55.08 ^b^	55.71 ^b^	94.45 ^a^

TE—trolox equivalents; ACI—antioxidant composite index; different letters in the same row indicate significant difference (*p* < 0.05).

**Table 7:** Antimicrobial activity of goji berry fruit extracts determined by the microdilution method.

Microorganisms	MIC						
	Red Goji	Yellow Goji	Black Goji	AM	COL	CIP	FCZ
	mg/mL			μg/mL			
**Gram-positive bacteria**							
*Staphylococcus aureus*	>2.0	>2.0	>2.0	4 (S)	N/A	0.001 (S)	N/A
*Staphylococcus epidermidis*	>2.0	>2.0	>2.0	8 (S)	N/A	0.001 (S)	N/A
*Enterococcus faecalis*	>2.0	>2.0	>2.0	N/A	N/A	2 (S)	N/A
**Gram-negative bacteria**							
*Escherichia coli*	>2.0	>2.0	>2.0	8 (S)	0.5 (S)	0.125 (S)	N/A
*Klebsiella pneumoniae*	>2.0	2.0	>2.0	4 (S)	1 (S)	0.25 (S)	N/A
*Salmonella abony*	2.0	2.0	>2.0	4 (S)	0.5 (S)	0.125 (S)	N/A
*Pseudomonas aeruginosa*	2.0	2.0	>2.0	16 (S)	1 (S)	0.001 (S)	N/A
**Yeast**							
*Candida albicans*	2.0	2.0	>2.0	N/A	N/A	N/A	2 (S)

MIC—minimum inhibitory concentration; AM—amikacin; COL—colistin; CIP—ciprofloxacin; FCZ—fluconazole; S—sensitive; N/A—not applicable.
